# Surgery to relieve nasal obstruction: outcome for 366 patients operated on by one senior surgeon

**DOI:** 10.1007/s00405-021-06696-7

**Published:** 2021-02-23

**Authors:** Lars Aksel Pedersen, S. Dölvik, K. Holmberg, C. Ahlström Emanuelsson, H. Johansson, L. Schiöler, J. Hellgren, S. Steinsvåg

**Affiliations:** 1 Department of Otorhinolaryngology, Head and Neck Surgery, Institute of Clinical Sciences, Sahlgrenska University Hospital, Sahlgrenska Academy, University of Gothenburg, Gröna Stråket 9, 413 45 Gothenburg, Sweden; 2 Agro ENT, Asker, Norway; 3 Department of Otorhinolaryngology, Head and Neck Surgery, Skane University Hospital, Lund University, Lund, Sweden; 4 Mary McKillop Institute for Health Research, Australian Catholic University, Melbourne, Australia; 5 Department of Occupational and Environmental Medicine, Sahlgrenska Academy, University of Gothenburg, Göteborg, Sweden; 6 Department of Otorhinolaryngology, Head and Neck Surgery, Sørlandet Hospital, Kristiansand, Norway; 7 Department of Otorhinolaryngology, Head and Neck Surgery, Haukeland University Hospital, Bergen, Norway

**Keywords:** General health, Nose VAS, Septoplasty, Turbinoplasty

## Abstract

**Background:**

Studies of patient-rated outcome in septoplasty and turbinoplasty most frequently involve several surgeons with varying surgical skills, techniques and experience. The aim of the present study was to evaluate outcome based on one experienced surgeon.

**Methods:**

Three hundred and sixty-six consecutive patients referred for nasal obstruction were included. All the patients were examined with nasal endoscopy before and after decongestion, they filled out a nose VAS and rated their overall general health before and three to six months after surgery. The patients underwent septoplasty, septoplasty plus turbinoplasty or turbinoplasty.

**Results:**

The mean nose VAS for nasal obstruction (0–100) preoperatively was 64.7 for all patients. Patients undergoing septoplasty (*n* = 159) were younger than patients undergoing septoplasty + turbinoplasty (*n* = 79) or patients undergoing turbinoplasty alone (*n* = 128). The nose VAS for nasal obstruction improved significantly in all three groups and 25% had a normal nose VAS after surgery in the septoplasty and septoplasty + turbinoplasty groups compared to only 8% in the turbinoplasty alone group. There was no significant difference in the improvement in nasal obstruction between septoplasty and septoplasty + turbinoplasty, but the septoplasty + turbinoplasty group experienced a significantly greater improvement in general health.

**Conclusions:**

In 366 patients operated on by one experienced surgeon, septoplasty and septoplasty + turbinoplasty were more effective at relieving nasal obstruction than turbinoplasty alone. Septoplasty + turbinoplasty resulted in a greater improvement in general health than septoplasty alone, despite the same improvement in nasal obstruction, indicating a beneficial effect of additional turbinoplasty in septoplasty.

## Introduction

Nasal obstruction is a common complaint in patients treated in ENT practice. A structural nasal obstruction often depends on a deviated nasal septum, enlarged turbinates or a combination of both. Septoplasty alone or in combination with turbinoplasty or turbinoplasty alone are the three most common surgical procedures for treating structural nasal obstruction. These surgical procedures are thus some of the most frequently performed in ENT departments worldwide, but predictors of outcome for one procedure compared with another are still poorly understood.

Studies evaluating the effect of septoplasty and turbinoplasty report varying results. In a review from 2018, patient satisfaction after septoplasty was between 50 and 100% after a minimum of 12 months postoperatively [1]. In two studies of large cohorts from the Swedish quality register for septoplasty, the surgical result was evaluated. In 5,865 patients from the original register, 76% rated their symptoms as “gone” or “almost gone” six months after surgery [2]. In a study from the new register, 63% of 888 patients reported an improvement of one level (for example, moderate to mild) in their self-rated nasal obstruction after 12 months [3]. Adding turbinoplasty to the septoplasty had no significant effect, even if this has been reported [4].

Most studies of the surgical outcome after septoplasty and turbinoplasty involve many different surgeons with varying skills, experience and surgical techniques. In a recent study by Nilsen et al. [5], nasal symptoms (VAS) and health-related quality of life (HrQol) were compared before and after (1) septoplasty alone, (2) septoplasty + radiofrequency therapy of the inferior turbinate (RFIT) and (3) RFIT alone. The study included 171 patients, operated on by 14 different surgeons at one hospital. Six months after surgery, the patients in Groups 1 and 2 experienced a significantly greater improvement in nasal patency than patients in Group 3, indicating that septoplasty is more effective than turbinoplasty alone, but also that turbinoplasty had no additional effect in septoplasty.

The inclusion of many different surgeons in a study introduces an inter-individual variation that could be substantial and could mask a true difference in surgical outcome between different surgical techniques. By studying the results for just one experienced surgeon, the inter-individual variation related to the surgeon is reduced. However, studies involving only one surgeon are rare in modern literature. Valsamidis et al. [6] performed a study which comprised 60 patients with nasal obstruction that were diagnosed with septal deviation. All the operations were performed by the same consultant surgeon and the study was designed to identify predictive factors that influenced the patients’ disease-specific HrQol six months postoperatively. Symptom severity as well as stress levels predicted overall HrQol after septoplasty + cauterisation of the inferior turbinate, using NOSE and SNOT 22 questionnaires. Patient-rated satisfaction in terms of the percentage of patients relieved of their nasal obstruction was not reported.

In the present study, we have included 366 consecutive patients that were referred for nasal obstruction. They were all diagnosed, and operated on, by the same ENT surgeon, with more than 20 years’ experience of septoplasty. To our knowledge, this is the largest cohort in which the effect of septoplasty alone, septoplasty plus turbinoplasty and turbinoplasty alone was compared, based on a single experienced surgeon’s practice.

## Materials and methods

### Study population

The study population comprises 366 adult patients (224 males and 142 females) with nasal obstruction diagnosed and operated on with septoplasty alone, septoplasty + turbinoplasty or turbinoplasty alone. All the patients were referred to the Agro ENT clinic in Asker, Norway, due to nasal obstruction by ENT specialists in the most densely populated south-eastern part of Norway.

A medical history was taken and the nose was examined with an endoscope before and after decongestion of the nasal mucosa with oxymetazoline (Nezeril^®^). Based on a total assessment, the surgeon decided to proceed with septoplasty alone, septoplasty + turbinoplasty, turbinoplasty alone or no surgery. A few patients were referred from other ENT specialists for turbinoplasty, which was re-assessed by the operating surgeon. Patients who were not recommended surgery or who did not want surgical treatment were not included in the study. Because this was a surgical centre, the majority of the patients fulfilled the criteria for surgery at this day surgery clinic. Reasons for not being recommended surgery at this clinic were cardiac disease, treatment with anticoagulants or the need for revision surgery. These patients were referred to a hospital.

Patients proceeding to surgery completed a questionnaire including questions about age, gender, allergy, asthma and smoking habits, as well as visual analogue scales (0–100) for sino-nasal symptoms (nose VAS). Postoperatively, the patients completed the same questionnaire. The time to follow-up ranged from three to six months when the patients again filled out the nose VAS for symptoms.

### Nose symptoms on visual analogue scales (nose VAS)

Patients put a mark on a 100 mm linear scale ranging from no symptoms to worst possible symptoms. The following symptoms were included; nasal obstruction, nasal discharge, oral breathing, snoring, sleep apnea, headache, midface pain, coughing, sneezing, sense of smell and sinusitis, as well as general health. The nose VAS has been used in other studies to assess nasal symptoms before and after nasal interventions and treatments [5–8]. In addition to its high sensitivity, reliability and reproducibility, this VAS is easy and simple to use for both patients and healthcare providers [8].

### Surgical procedures

#### Septoplasty

Septoplasty was performed under local anaesthesia with mild sedation (adrenalin-tetracaine on sponges for 20 min, injections with 1% Xylocaine^®^ adrenalin (Astra-Zeneca^®^) into the septum and 25–25 mg fentanyl (Sandoz^®^) and 2.5–5 mg stesolid (Actavis^®^) iv. respectively) and all the patients had a right-sided hemi-transfixion incision. Upper and lower tunnels were established bilaterally. Depending on the underlying septal pathology, the septum was straightened and basal cristae were resected. When needed, septal cartilage was taken out and re-implanted after being straightened. Postoperatively, bilateral Teflon stents were applied for seven days, with spongostan dressings for 24 h.

### Turbinoplasty

Depending on the underlying pathology, the inferior turbinates were reduced in size using coblation (mucous membrane oedema), lateralised and/or had a minor inferior-lateral resection (enlarged concha bone) under local anaesthesia with adrenalin-tetracaine on sponges for 20 min and injections with 1% Xylocaine^®^ adrenalin (Astra-Zeneca^®^) into the turbinates. Postoperatively, merocel dressings were applied for 48 h.

The study group was originally meant to be a local quality register. However, discussion between colleagues disclosed scientific potential that warranted further exploration. The study was approved by the National Ethics Committee of Norway (reference number 134609) and investigations were performed in accordance with the principles of the Declaration of Helsinki/Hong Kong.

### Statistics

When describing the study population, comparisons between the three groups with different surgical procedures were made using Fisher’s exact test and Fisher’s permutation test. When studying the change after surgery, Fisher’s test for pairwise comparisons was used. Two-sided tests were used and *p* < 0.05 was considered statistically significant.

## Results

Baseline data for the study population of 366 patients are shown in Table 1. The mean age was 39.1 years. Allergy, asthma and smoking habits were similar to those of the general Norwegian population [9, 10]. Preoperatively, the nose VAS was highest for nasal obstruction and mouth breathing, Table 2. The greatest improvement on the VAS was seen in these two groups, followed by snoring, nasal discharge and reduced sense of smell, Table 2. All nose VAS symptoms improved significantly after surgery, significantly and representing a relevant clinical difference.

**Table 1:** Age, gender, allergy, asthma and smoking in all subjects and in surgical subgroups at baseline

	All (*n* = 366)	Septoplasty + turbinoplasty (*n* = 79)	Septoplasty (*n* = 159)	Turbinoplasty (*n* = 128)
Age, years, mean (SD)	39.1 (13.9)	42.7 (11.9)	36.4 (13.4)*	40.4 (14.9)
Female gender, %	39	49**	33	39
Allergy, %	39	37	39	40
Asthma, %	9	11	7	9
Smokers, %	21	20	22	20
How many cigarettes, mean (SD)	1.7 (4.6)	1.6 (4.9)	2.1 (5.0)	1.3 (3.9)
How many years, mean (SD)	4.3 (9.6)	4.3 (9.8)	4.2 (9.2)	4.5 (10.2)
BMI kg/m^2^, mean (SD)	24.8 (3.6)	24.5 (3.3)	25.0 (3.3)	24.9 (4.2)

Significant differences are marked with * and **

^*^Septoplasty vs septoplasty + turbinoplasty *p* < 0.001, septoplasty vs turbinoplasty *p* = 0.019

^**^Septoplasty + turbinoplasty vs septoplasty *p* = 0.025

**Table 2:** Nose VAS symptoms (0–100), mean (SD) for all included patients, *n* = 366 at baseline, as well as change 3–6 months after surgery

Nose VAS	*N*	Baseline, mean (SD)	Change after surgery, mean (SD)	Change %	*p* value
Nasal obstruction	366	64.7 (20.2)	− 36.8 (26.9)	57	< 0.001
Mouth breathing	364	58.0 (27.6)	− 24.2 (30.5)	42	< 0.001
Snoring	358	49.9 (32.2)	− 16.8 (26.5)	34	< 0.001
Apneas	287	26.0 (29.2)	− 10.0 (27.8)	38	< 0.001
Nasal discharge	362	46.5 (28.8)	− 17.0 (30.0)	37	< 0.001
Headache	366	35.3 (28.0)	− 13.6 (25.2)	38	< 0.001
Facial pain	363	21.5 (25.7)	− 9.0 (24.4)	42	< 0.001
Sinusitis	357	29.7 (28.1)	− 14.9 (25.9)	50	< 0.001
Cough	363	30.1 (24.7)	− 9.4 (23.7)	31	< 0.001
Sneezing	365	42.2 (25.9)	− 9.8 (26.4)	23	< 0.001
General health	362	26.9 (25.8)	− 10.2 (25.0)	38	< 0.001
Reduced sense of smell	362	40.5 (30.5)	− 16.5 (28.0)	41	< 0.001

Of the 366 patients, 79 had a septoplasty + turbinoplasty, 159 had a septoplasty alone and 128 a turbinoplasty. A comparison between the three different surgical interventions revealed no significant differences on the nose VAS for nasal obstruction preoperatively, regarding asthma, allergy, smoking habits or BMI (Table 1). Patients undergoing septoplasty (n = 159) were younger than patients undergoing septoplasty + turbinoplasty (n = 79) or turbinoplasty alone (n = 128). The nose VAS for nasal obstruction improved significantly in all three groups, Table 3. A total of 25% had a normal nose VAS (< 9) after septoplasty alone and septoplasty + turbinoplasty, compared with only 8% in the turbinoplasty group. Patients undergoing a turbinoplasty alone had more nasal obstruction postoperatively compared with the other groups. There was no significant difference in the improvement in nasal obstruction between septoplasty alone and septoplasty + turbinoplasty. Surgery, including all three procedures, had a significant effect on all symptoms. General health improved the most in patients undergoing septoplasty + turbinoplasty, followed by septoplasty, Table 3.

**Table 3:** Nose VAS symptoms (0–100), mean (SD) for the three different surgical groups, septoplasty + turbinoplasty, septoplasty alone, turbinoplasty alone at baseline and change from baseline after surgery, as well as change in %

	Septoplasty + turbinoplasty (*N* = 79)				Septoplasty (*N* = 159)				Turbinoplasty (*N* = 128)				
	Baseline—change after surgery				Baseline—change after surgery				Baseline—change after surgery				
	Mean (SD)	Mean (SD)	%	*p* value	Mean (SD)	Mean (SD)	%	*p* value	Mean (SD)	Mean (SD)		%	*p* value
Nasal obstruction	63.3 (21.7)	− 42.4 (26.5)	67	< 0.001	65.5 (20.3)	− 40.5 (25.7)	62	< 0.001	64.4 (19.3)	− 28.7 (27.0)	45		< 0.001
Mouth breathing	57.8 (26.5)	− 29.7 (31.1)	51	< 0.001	62.2 (26.8)	− 28.2 (30.3)	45	< 0.001	52.8 (28.6)	− 15.7 (28.6)	30		< 0.001
Snoring	45.3 (31.8)	− 20.7 (23.9)	46	< 0.001	52.3 (31.9)	− 18.5 (27.0)	35	< 0.001	49.5 (32.9)	− 12.6 (26.8)	25		< 0.001
Apneas	18.8 (22.9)	− 7.2 (21.4)	38	0.019	32.2 (31.2)	− 15.8 (27.9)	49	< 0.001	22.9 (28.6)	− 4.4 (29.5)	19		0.16
Nasal secretion	60.8 (27.1)	− 31.8 (27.6)	52	< 0.001	45.9 (27.9)	− 17.7 (30.9)	39	< 0.001	38.4 (27.8)	− 7.0 (26.4)	18		0.0036
Headache	43.0 (29.2)	− 24.3 (24.9)	57	< 0.001	33.9 (26.7)	− 14.0 (25.2)	41	< 0.001	32.4 (28.0)	− 6.7 (22.9)	21		0.0011
Facial pain	32.7 (28.6)	− 19.8 (29.7)	60	< 0.001	20.4 (24.6)	− 8.5 (24.4)	42	< 0.001	16.0 (23.0)	− 3.2 (18.2)	20		0.047
Sinusitis	48.3 (29.7)	− 31.3 (29.3)	65	< 0.001	23.9 (26.0)	− 11.1 (24.8)	46	< 0.001	25.6 (24.6)	− 10.0 (20.8)	39		< 0.001
Cough	36.8 (27.4)	− 18.0 (23.7)	49	< 0.001	30.4 (22.9)	− 9.1 (24.0)	30	< 0.001	25.5 (24.3)	− 4.5 (22.1)	18		0.022
Sneezing	47.1 (27.3)	− 15.4 (27.4)	33	< 0.001	42.8 (25.1)	− 9.5 (27.5)	22	< 0.001	38.3 (25.8)	− 6.8 (23.9)	18		0.0016
General health	37.7 (27.3)	− 20.9 (29.1)	55	< 0.001	24.4 (24.1)	− 10.5 (23.8)	43	< 0.001	23.2 (25.3)	− 3.1 (21.1)	13		0.11
Reduced sense of smell	55.4 (29.1)	− 30.9 (31.5)	56	< 0.001	37.3 (30.1)	− 16.9 (27.4)	45	< 0.001	35.1 (29.1)	− 6.8 (22.0)	19		< 0.001

**Fig. 1:**
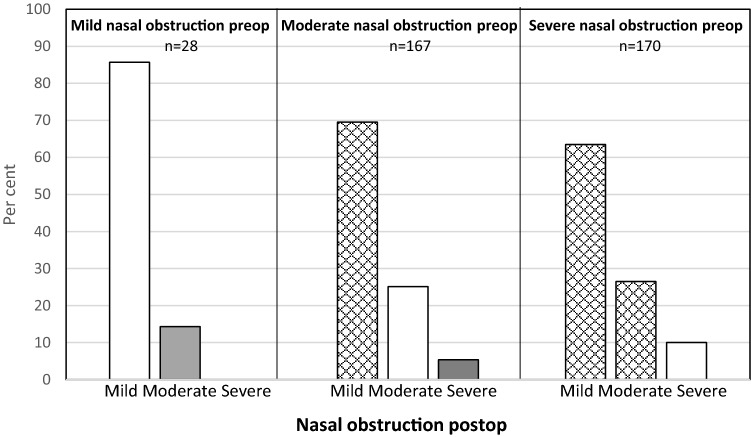
Change in self-rated nose VAS for nasal obstruction after surgery for all three procedures (septoplasty + turbinoplasty, septoplasty alone, turbinoplasty alone). Checked: improved, white: unchanged, grey: deterioration

After dividing the patients’ nasal obstruction into three degrees: mild (VAS < 30), moderate (VAS 30–70) and severe (VAS > 70), the patients improved as described in Fig. 1. Improvement was defined as moving to a less severe degree. Patients with severe nasal obstruction had the greatest chance of improvement, whereas those with mild nasal obstruction had the least.

**Fig. 2:**
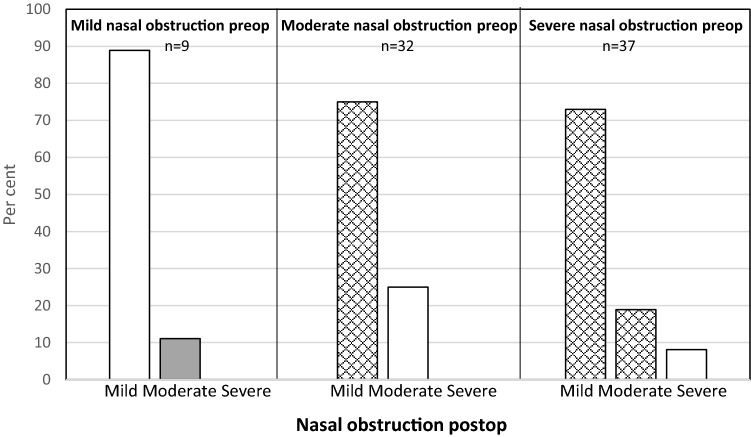
Change in self-rated nose VAS for nasal obstruction after septoplasty + turbinoplasty. Checked: improved, white: unchanged, grey: deterioration

**Fig. 3:**
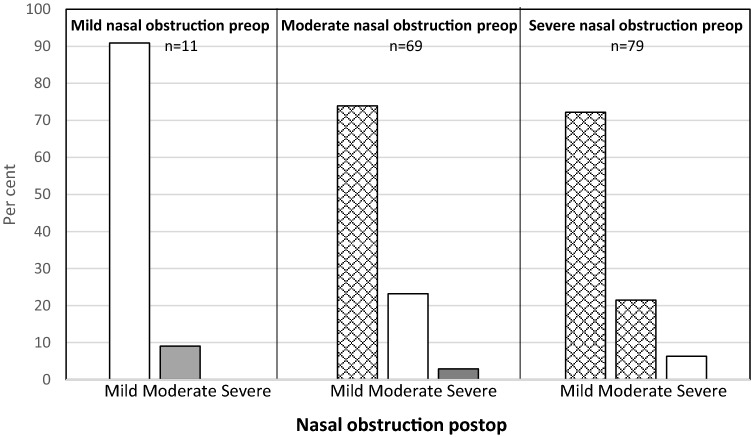
Change in self-rated nose VAS for nasal obstruction after septoplasty alone. Checked: improved, white: unchanged, grey: deterioration

**Fig. 4:**
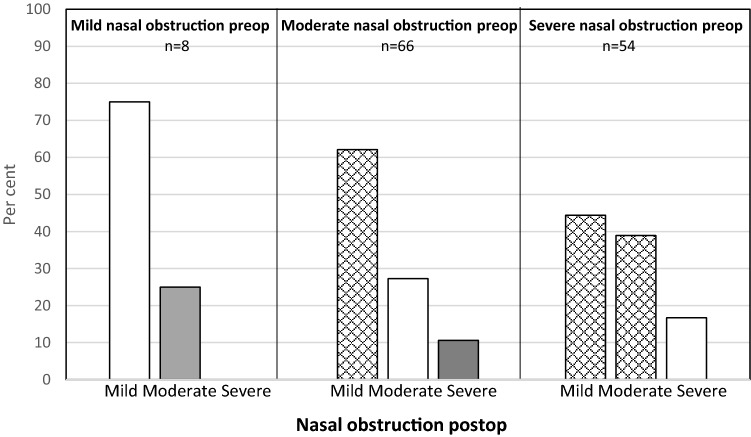
Change in self-rated nose VAS for nasal obstruction after turbinoplasty alone. Checked: improved, white: unchanged, grey: deterioration

## Discussion

In this study of 366 patients with structural nasal obstruction, diagnosed and operated on by the same experienced ENT surgeon, there was a significantly greater improvement in general health after septoplasty + turbinoplasty than after septoplasty alone, indicating that additional turbinoplasty in septoplasty is beneficial for the overall surgical result. Turbinoplasty alone was, however, less effective than septoplasty + turbinoplasty and septoplasty alone.

Over the years, many surgical techniques have been described when it comes to relieving nasal obstruction by surgery on the septum and the lateral wall of the nasal cavity, including the classic Cottle and Killian techniques. The actual performance of septoplasty and turbinoplasty may therefore vary considerably between ENT surgeons, as the individual implementation of techniques and surgical instruments is common. The situation is further complicated by the lack of standardisation in the diagnostic procedure and the decision-making of patients undergoing nasal surgery, leaving a great deal of room for the personal interpretation of symptoms and clinical findings between different surgeons. Many studies of septoplasty and turbinoplasty bypass this problem by simply referring to the surgical procedure by name. In this study, only one experienced surgeon diagnosed all the patients and decided which surgical procedure to perform, as well as performing all the surgical procedures. We believe that this considerably reduced the variation in the diagnosis of the patients, the patient selection for the specific surgical procedures and the performance of the actual surgery.

The nose VAS is a validated tool for assessing nasal symptoms that correlates well with objective measurements of nasal obstruction, such as acoustic rhinometry and PNIF^5,11^. Independent of surgical procedure, these 366 patients experienced a mean relief of nasal obstruction of 36.8 on the nose VAS (0–100), corresponding to a 57% improvement after three to six months. This means that symptoms were reduced by more than half and that the postoperative mean nose VAS for nasal obstruction was 30. Rhee et al. regard a change of 30 on the VAS as a clinically meaningful measurement of success [11]. It also means that the patients did not fully return to normal nasal patency. In this study, 25% of the patients had a postoperative normal, < 9, nose VAS for both the septoplasty alone and septoplasty + turbinoplasty group, compared with only 8% in the turbinoplasty alone group [12]. Rhee et al. found that the average VAS score for individuals with no nasal airway obstruction was around 20 [11]. This may indicate that our limit for a normal nose of < 9 VAS is low.

When dividing all the patients into three groups with regard to nasal obstruction, mild (VAS < 30), moderate (VAS 30–70) and severe (VAS > 70), most of the patients improved, defined as moving to a group with less severe symptoms after surgery, Fig. 1. For the patients with moderate nasal obstruction, almost 70% improved to mild. In the severe group, 90% improved to either mild or moderate. This is a better result than that observed in the study from the Swedish National Septoplasty Register based on a large number of procedures performed by many different ENT surgeons [3]. Figures 2, 3 and 4 show that septoplasty and septoplasty + turbinoplasty result in a greater improvement than turbinoplasty alone.

In patients with allergic rhinitis, nasal obstruction is regarded as the most troublesome symptom and it has also been strongly associated with poor sleep [13]. Nasal surgery in the present study also had an effect on other symptoms, such as mouth breathing, which was reduced by 42%, confirming a clinical effect on nasal patency. The positive effect on snoring, nasal secretion, sneezing and reduced sense of smell may be related to an improved local environment in the nasal cavity, involving the respiratory mucosa and mucociliary transport, when the obstruction is relieved and nasal breathing is normalised.

When comparing the effect on nasal obstruction between the three different surgical techniques, septoplasty + turbinoplasty showed a reduction of 67%, septoplasty alone of 62% and turbinoplasty of 45%. This study was not randomised between the surgical procedures. At baseline, there were no significant differences in preoperative mean nose VAS scores for nasal obstruction between the groups and the potential for improvement between the groups was therefore similar. In spite of this, the turbinoplasty group displayed the smallest improvement in nasal obstruction after surgery. The effect on mouth breathing was also smaller than after septoplasty, confirming a lower effect on nasal patency. It is also striking that 20% of the subjects in the group undergoing turbinoplasty alone with severe symptoms preoperatively reported severe nasal obstruction after surgery. In the two septoplasty groups, this was less than 10%. The results indicate that turbinoplasty alone is less effective in reducing structural nasal obstruction, despite the fact that it does result in a net increase in nasal cavity space, in comparison with septoplasty that “only” relocates space from one side to the other. It is also noteworthy that the patients undergoing turbinoplasty experienced no significant improvement in their general health. Turbinoplasty as a single procedure is used as a last resort when anti-inflammatory medical treatment is insufficient or failing and this was also the case in some of the patients in this study. The finding that turbinoplasty had the smallest effect on the nose VAS in this material is in accordance with the study by Nielsen et al. [5].

Septoplasty alone and septoplasty + turbinoplasty were the two most effective surgical procedures for relieving nasal obstruction in this study. There was no significant difference between them on the nose VAS for nasal obstruction. Septoplasty + turbinoplasty, however, had twice as large an effect on general health as septoplasty alone, indicating that the addition of turbinoplasty to septoplasty can have a positive effect.

Among the patients undergoing a septoplasty, there were more men and they were younger than patients undergoing a septoplasty + turbinoplasty and turbinoplasty alone. The predominance of men in septoplasty has been likened to more nasal trauma in this group related to sports activities, assault and motor vehicle accidents, among others [14]. Why isolated septal deviations underwent surgery at an earlier age is not clear, but it is important in the assessment, as surgery at a young age is associated with a risk of a poorer outcome [2].

Our results are comparable to those in the study by Nilsen et al. [5] and show that nasal surgery is effective in relieving structural nasal obstruction, but turbinoplasty alone is the least effective. In the Nilsen study, however, 171 patients were included compared with 366 in this study. The number of surgeons was 14 compared with one in this study. The surgery was performed at a university hospital under both general and local anaesthesia, indicating that the patients had a more severe disease and included 38 revision cases compared with none in this study. It is therefore interesting that the general health was more improved to a significant degree in septoplasty + turbinoplasty compared with septoplasty alone in this study but not in the study by Nielsen et al. This could be attributed to the larger number of surgeons involved, as well as the other heterogeneities in that study regarding patient selection. Other studies have also failed to show a clinically relevant difference when adding turbinoplasty to septoplasty in nasal obstruction [15]. In spite of this, many patients undergo the combined procedure and, in this study, these patients represented 22%. The effect on general health was both significant and clinically relevant, indicating that the addition of a turbinoplasty in selected patients is clearly beneficial. Standardising surgical outcome measurements, looking at single surgeons with known experience in control of both patient selection and the surgery itself, may thus be necessary to identify differences in nasal surgery based on patient phenotypes.

Nasal assessment should always be performed before and after nasal decongestion to exclude inflammatory swelling of the nasal mucosa as the cause of nasal obstruction. The nasal cavities should also be inspected with an endoscope to exclude polyps or tumours as the main cause of nasal obstruction. This was done in the present study.

This study has several limitations and the most obvious is that the patients were not randomised to the different treatments. The randomisation of patients with different and sometimes complicated diseases in terms of flow mechanics and intranasal geometry would probably not have been ethical and was not used. Another limitation of the study is that one single surgeon made the decision to operate and what surgery to perform based on his clinical examination and his 20 years of experience. This could therefore be described as a “real life study” and no control group was used. However, the fact that the overall results are comparable to those in the study by Nielsen et al., with a similar design but including 14 surgeons, strengthens the validity of the present results. The outcome of nasal surgery relies on both surgical skills and surgical techniques. The surgical techniques in this study were not formally standardised or validated.

In this study, we worked with a 10% improvement in VAS as statistically and clinically significant. We were unable to reach consensus on this and clinical significance in particular is a complicated question. A 10% improvement probably does not mean the same to a patient grading his or her VAS score as 90 as it does to another patient grading 30. In response to an article on a different field of medicine [16], there is a discussion about 10% and clinical significance, where the authors think this is a relevant limit. In another set of material using VAS for grading pain, the study group was divided into three different categories on the VAS scale (< 30, 31–70, > 70) [17], as we also did. They found that this was associated with clinical relevance. The same grading has previously also been used in material relating to the nose VAS [18].

## Conclusion

In 366 patients operated on by one experienced surgeon, septoplasty alone and septoplasty + turbinoplasty were more effective in relieving nasal obstruction than turbinoplasty alone. Septoplasty + turbinoplasty resulted in a greater improvement in general health than septoplasty alone, despite the same improvement in nasal obstruction, indicating a beneficial effect of additional turbinoplasty in septoplasty.
